# Convergent gut microbial functional strategies drive energy metabolism adaptation across Ursidae species and challenge the uniqueness of giant panda

**DOI:** 10.1093/ismejo/wraf201

**Published:** 2025-09-03

**Authors:** Tingbei Bo, Xiaoming Xu, He Liu, Liqiu Tang, Haihong Xu, Siqi Zhao, Jinzhen Lv, Dehua Wang

**Affiliations:** School of Grassland Science, Beijing Forestry University, Beijing 100091, China; State Key Laboratory of Animal Biodiversity Conservation and Integrated Pest Management, Institute of Zoology, Chinese Academy of Sciences, Beijing 100101, China; Beijing Key Laboratory of Captive Wildlife Technology, Beijing Zoo, Beijing 100044, China; State Key Laboratory of Animal Biodiversity Conservation and Integrated Pest Management, Institute of Zoology, Chinese Academy of Sciences, Beijing 100101, China; Beijing Key Laboratory of Captive Wildlife Technology, Beijing Zoo, Beijing 100044, China; Beijing Key Laboratory of Captive Wildlife Technology, Beijing Zoo, Beijing 100044, China; State Key Laboratory of Animal Biodiversity Conservation and Integrated Pest Management, Institute of Zoology, Chinese Academy of Sciences, Beijing 100101, China; School of Life Science, Shandong University, Qingdao 266237, Shandong, China

**Keywords:** Ursidae, gut microbiota, FMT, energy metabolism, season

## Abstract

The gut microbiota is a key regulator of host energy metabolism, but its role in seasonal adaptation and evolution of bears is still unclear. Although giant pandas are considered an extraordinary member of the Ursidae family due to their specialized herbivory and low metabolic rate, there is still controversy over whether the metabolic regulation mechanism of their gut microbiota is unique. This study analyzed the seasonal dynamics of gut microbiota in giant pandas (*Ailuropoda melanoleuca*), Asian black bears (*Ursus thibetanus*), brown bears (*Ursus arctos*), and polar bears (*Ursus maritimus*), and combined with fecal microbiota transplantation (FMT) experiments, revealed the following findings. The microbial composition of the four bear species is similar, with both *Firmicutes* and *Proteobacteria* dominating. The enrichment of *Firmicutes* in winter enhances lipid metabolism, and adapts to dietary differences, indicating the existence of convergent microbial functional strategies in the Ursidae family. Our results demonstrate that bear gut microbiota promoted seasonal adaptation. In FMT experiments, bear gut microbiota in winter may had stronger functional capabilities on regulating host energy metabolism in mice, and regulate host appetite to increase energy intake. Finally, despite feeding on bamboo, giant pandas microbiota driven energy metabolism pathways (such as SCFAs) are highly conserved compared to other bears, suggesting a deep commonality in the adaptability of bear microbiota in evolution. Therefore, this study challenges the traditional view of microbial uniqueness of giant pandas, and emphasizes the co-evolutionary mechanism of energy metabolism adaptation in bear animals through microbial plasticity. In the future, it is necessary to integrate wild samples to eliminate the interference of captive diet and further analyze the genetic basis of host gut microbiota interactions.

## Introduction

Seasonal fluctuations in mammalian energy metabolism are adaptive responses to cyclic environmental resource variations, involving dynamic equilibria between food availability and energy allocation (e.g. thermoregulation and reproduction). Ursidae, as a lineage with diversified energy strategies, exhibit metabolic patterns tightly linked to ecological behaviors. Active species (e.g. brown bears, and Asian black bears) require sustained high-energy intake due to elevated activity levels, whereas low-metabolism species (e.g. giant pandas) adapt to resource constraints through behavioral adjustments (e.g. reduced locomotion) [1]. Current studies reveal two core strategies for ursids to cope with energy stress in the cold environment. Hibernation-type: brown bears and black bears accumulate fat via hyperphagia in summer and reduce metabolic rates (body temperature drops by 30%–50%) during hibernation to conserve energy [2, 3]. This process involves phase-specific activation of lipogenesis-related genes to ensure efficient lipid utilization [4]. Continuous metabolism-type: non-hibernators like polar bears rely on insulated fur to minimize heat loss [5], whereas giant pandas maintain energy homeostasis by enhancing thermogenesis (e.g. *TRPM8* inhibition-mediated cold tolerance) and microbial-driven cellulose fermentation [6]. Giant pandas lack endogenous cellulase genes [7] but depend on gut microbiota for bamboo fiber degradation [8], suggesting microbial compensation in energy provision.

The gut microbiota, acting as a “second genome” for host metabolism, responds to environmental fluctuations through pathways such as short-chain fatty acid (SCFA) synthesis and lipid metabolism regulation [9]. For instance, hibernation-associated gut microbiota remodeling in brown bears sustains host lipid homeostasis [4], whereas seasonal dietary shifts (carnivory to herbivory) in polar bears significantly alter microbial composition [10]. However, current research on ursid microbial seasonal adaptation primarily focuses on hibernators, leaving the microbial dynamics and evolutionary implications in non-hibernating species (e.g. giant pandas) poorly understood.

Ursids display remarkable diversity in diet, habitat, and seasonal behaviors, with energy demands varying drastically from the carnivorous polar bear to the herbivorous giant panda. We hypothesize that ursids may adapt to energy challenges through convergent gut microbial functional modules (rather than species-specific taxa) and that host phylogeny predominates over diet or seasonal factors in shaping metabolic phenotypes. To test this, we systematically analyzed seasonal gut microbiota dynamics in four ursids (polar bear, giant panda, brown bear, and Asian black bear) and conducted fecal microbiota transplantation (FMT) experiments to dissect their metabolic regulatory mechanisms. The study addresses three key questions: Do gut microbiota of ursids with divergent diets share seasonal response patterns? How does the microbiota mediate host energy allocation strategies (e.g. thermogenesis vs. storage)? Does the energy metabolism driven by gut microbiota in giant pandas have uniqueness?

## Material and methods

### Animals

Four bear species were included in this study, comprising giant pandas, polar bears, black bears, and brown bears. Comprehensive information for each bear is detailed in Table S1. They were housed in Beijing Zoo, where they were managed under consistent conditions, including diet and housing. Seasonal supplements, including vegetable and fruits were also provided based on the primary nutritional ingredient. The primary diets were presented in Table S2.

We designed a transplantation experiment to verify the functionality of the fecal microbiota. In this experiment, sixty one BALB/c mice were used as the receptor animals. Two-month-old mice were purchased from SPF Biotechnology Co., Ltd. (Beijing, China). The mice were individually housed in plastic cages under controlled conditions: room temperature (23 ± 1°C) and a photoperiod of 16 hours light: 8 hours dark. Following the transplantation experiment, the mice were euthanized using carbon dioxide, the feces and tissue samples (hypothalamus, brown adipose tissue, small gut, liver) were collected and immediately frozen at −80°C for preservation.

### Fecal sample collection

Fecal samples were collected from bears housed at the Beijing Zoo across all four seasons. The species included giant pandas, polar bears, brown bears, and black bears. Sampling was conducted in April (spring), July (summer), October (autumn), and January (winter), with fecal samples collected separately for each of the four bear species during these months. The keeper waits for the bear to defecate after feeding, collects large particles of feces in sterile cloth, opens the feces with sterilized scissors, and uses a sterile spoon to scoop out 10–20 g of feces in the middle and place it in a sterile centrifuge tube. All stool samples (a total of 81) were immediately frozen and stored at −80°C for preservation.

### Fecal microbiota transplant

To deplete the gut microbiota, mice were administered a fresh cocktail of antibiotics via intragastric gavage (200 μl/day) for 6 consecutive days. The antibiotic cocktail contained neomycin (100 μg/ml), streptomycin (50 μg/ml), and penicillin (100 U/ml, Sigma, Germany) [11]. For microbiota transplantation, frozen fecal samples collected from bears during summer and winter were diluted in 0.9% normal saline. Specifically, 200 mg of feces was suspended in 2 ml of saline, and a 200 μl aliquot of the suspension was administered via intragastric gavage to each microbiota-depleted recipient mouse. Once a day, lasting for 21 days. Winter fecal transplantation group: Panda-FMT(W), Polar-FMT(W), Black-FMT(W), and Brown-FMT (W). For the control group, 200 μl of normal saline was administered to each mouse (n = 5). Summer fecal transplantation group: Panda-FMT(S), Polar-FMT(S), Black-FMT(S), Brown-FMT(S), and a control group (n = 7).

### Measurement of body weight of mice

Body weight of the mice were measured at 9 a.m. every 2 days, using an electronic balance (Sartorius Model BL 1500 ± 0.1 g).

### Measurement of resting metabolic rate of mice

Resting metabolic rate (RMR) was estimated by measuring the rate of oxygen consumption using an open-flow respirometry system (TSE Systems GmbH, Germany) at the end of the FMT. The data were recorded and averaged every 10 s by a computer connected via an analogue-to-digital converter that converted the changes of air composition to digital signal, then analyzed using Labmaster software. The mice had access to food and water prior to the measurements of RMR, during which subjects were deprived of food and water. RMR was measured for 3 hours at 30 ± 0.5°C and calculated from the lowest rate of oxygen consumption over 10 min. RMR measurements for all subjects were completed during a 2-day window [12].

### 16S rRNA gene sequencing analysis

Bacterial DNA was extracted from the fecal samples of bears and mice using the DNeasy PowerSoil Kit (Qiagen, Hilden, Germany). The V3-V4 hypervariable regions of the bacterial 16S rRNA gene were amplified via PCR using universal primer pairs (343F: 5F: 56S g the DNeasy PowerSoil Kit (Qiagen, Hilden, Germany). Thd water. RMR was meMiSeq System (Illumina) with paired-end read cycles of 300 bases each (OE Biotech, Shanghai, China). The raw sequencing data were processed as follows: Primer sequences were trimmed from the raw data using Cutadapt software [13]. Quality filtering, noise reduction, splicing, and chimera removal were performed using DADA2 within QIIME2 (version 2020.11) with default parameters [14]. We used the SILVA rRNA database (v138.2) for taxonomic classification of quality-filtered ASVs (https://ftp.arb-silva.de). Species annotation was performed using QIIME2 software. To study the phylogenetic relationship of each ASV, and the differences of the dominant species among different samples (groups), multiple sequence alignment was performed using QIIME2 software. The absolute abundance of ASVs was normalized using a standard sequence number, corresponding to the sample with the least sequences. Subsequent analyses of alpha diversity and beta diversity were all performed based on the output normalized data. Alpha-diversity index (Shannon index) was calculated by mothur software (version 1.30). To find out the significantly different species at each taxonomic level (phylum class order family genus species), the R software (Version 3.5.3) was used to do MetaStat and Kruskal-Wallis test analysis. Principal Coordinate Analysis (PCoA) based on Bray-Curtis metrics were used to visualize the structure of microbial community (beta diversity). The significance for PCoA analyses was detected with multivariate permutation tests using the nonparametric method ANOSIM (permutations = 999) included in the R “vegan” package. The linear discriminant analysis (LDA) Effect Size (LEfSe) method was used to assess differences in microbial communities using a LDA score threshold of 2 [15]. KEGG (Kyoto Encyclopedia of Genes and Genomes) pathways overrepresented in the gut microbiome were predicted using PICRUSt2 based on several gene family databases.

### Transcriptome sequencing and analysis

Total RNA was extracted from mice liver tissues using the TianGen RNA Easy Fast Animal Tissue/Cell Total RNA Extraction Kit. Mice intestinal transcriptome samples were sequenced using the MGI-2000 sequencing platform, employing paired-end PE150 sequencing for eukaryotic transcriptomes (Wekemo Tech Group, Shenzhen, China). Following sequencing, the raw data underwent quality control and filtering to obtain clean reads for subsequent analysis. Gene expression levels in the intestinal tissues of mice were quantified using STAR+RSEM, with expression levels normalized using FPKM (Fragments Per Kilobase per Million fragments). Differential gene expression analysis was performed using DESeq2, and significantly differentially expressed genes (DEGs) were identified based on the criteria of |log2 fold change| > 1 and *P* value <0.05. To further explore the biological significance of these DEGs, functional enrichment analysis was conducted using KOBAS 3.0. This included Gene Ontology and KEGG pathway analyses. These analyses identified significantly enriched functional pathways and highlighted the key biological functions associated with the differentially expressed genes.

### Measurement of protein expression by western blot

Tissue samples of the Brown adipose tissue (BAT) and small gut were weighed, and homogenized in 200 μl of radioimmunoprecipitation assay buffer. Homogenates were centrifuged at 16 200 g at 4°C for 30 min. Protein from the supernatant was placed in loading buffer and denatured by heating at 100°C for 3 min. Total protein was separated by SDS-PAGE using a Mini Protean apparatus (BioRad Laboratories, PA, USA) then transferred to PVFD membranes. Membranes were incubated for 12 hours at 4°C or 2 hours at room temperature in 5% skim milk powder to reduce nonspecific antibody binding. The membranes were then exposed to primary antibodies (below) for >12 hours at 4°C. Then, membranes were exposed to appropriate secondary antibodies for 2 hours at room temperature (either peroxidase-conjugated goat anti-rabbit IgG (111–035-003, Jackson, USA) or peroxidase-conjugated goat antimice IgG (115–035-003, Jackson) depending on the primary antibody). Reaction products were visualized by chemiluminescence (ECL, Yesen, China). Protein was quantified with Lab image Software (BioRad Laboratories), expressed as relative units to housekeeping proteins.

Primary antibodies: anti-uncoupling protein 1 (UCP1, ab155117, Abcam, UK), anti-peroxisome proliferator-activated receptor gamma coactivator-1 alpha (PGC1α, ab188102, Abcam), anti-tyrosine hydroxylase (TH, AB152, Merck Millipore, Germany), anti-free fatty acid receptor 2 (FFAR2, ABC299, Merck Millipore), anti-cAMP Protein Kinase Catalytic subunit antibody (cAMP, ab26322, Abcam), anti-monocarboxylic acid transporter 1 (MCT1, ab93048, Abcam), GAPDH (A01020, Abbkine, China) as a housekeeping protein.

### Total RNA extraction and qPCR analysis of hypothalamus

Total RNA was extracted from the hypothalamus using TRIzol reagent, following which reverse transcription was performed to generate cDNA according to the supplier’s specifications (Code No. RR820Q/A/B, TAKARA, Dalian, China). For RT-qPCR analysis, 2 μl of the generated cDNA was used as a template for subsequent PCR reactions with gene-specific primers (Table S3). The final reaction volume of 20 μl contained 10 μl of 2ained 10 ted cDNA was used as μl of 50 × ROX Reference Dye, 2 μl cDNA template, 0.8 μl of forward primer and reverse primer (final concentration 0.4 μM per primer), and 6 μl dH_2_O. The RT-qPCR was conducted using PikoReal Software 2.2 on a PikoReal 96 instrument (Thermo Scientific, USA). After an initial polymerase activation step at 95°C for 60 s, amplification was followed by 40 cycles (95°C for 5 s, 55°C for 30 s, and 72°C for 30 s). The reaction was finished by the built-in melting curve. All samples were quantified for relative quantity of gene expression by using actin expression as an internal standard. The expression levels of appetite-related neuropeptides were analyzed, including Neuropeptide Y (NPY), Agouti-related protein (AgRP), Pro-opiomelanocortin (POMC), Cocaine- and amphetamine-regulated transcript (CART).

### Measurement of SCFAs via gas chromatography

The concentrations of SCFAs, specifically acetic acid, propionic acid, and butyric acid, of bear feces and mouse cecal contents were measured using gas chromatography (GC) (Agilent 7890 A, Agilent Technologies, Germany) according to a previously described protocol [16]. The SCFAs were separated in a 30 m × 0.25 mm × 0.25 μm DB-WAX column (polyethylene glycol 20 000, Agilent Technologies). The system was operated at a maximum temperature of 250°C with helium (> 99.999%) as a carrier gas at a constant flow rate of 1 ml/min. Splitless injection of 0.5 μl samples was performed at 230°C. The temperature was programmed at 60°C for 1 min, increased at a rate of 5°C/min to 200°C, and then increased at 10°C/min to 230°C. The total running time lasted 32 min for each sample. The SCFAs were identified by comparing their retention times with those of authentic reference compounds and were quantified based on the abundance relative to that of the standard.

### Statistical analysis

TimeTree (http://www.timetree.org) was used to build a phylogenetic tree with a timeline for these four species of bears [17, 18]. TimeTree is a public knowledge base that provides information on the evolutionary timescale of life. Data from thousands of published studies are assembled into a searchable tree of life scaled to time. Statistical analysis was conducted using the SPSS 22.0 software package and GraphPad Prism 9. Differences in body mass was compared between treatment groups using a repeated-measure ANOVA. Measurements of other indexes were compared using one-way or two-way ANOVA followed by Tukey’s multiple comparisons test. Inter group comparison of microbial abundance were using Kruskal-Wallis test and Mann–Whitney U test. Spearman correlation were used to calculate the correlation between specific genus and physiological measurements. Data are presented as the mean ± standard error of the mean (SEM), and *P* < .05 was considered a statistically significant difference. (^*^P < .05, ^**^P < .01, ^***^P < .001, ^****^P < .0001).

## Results

### Interspecific variation in bear gut microbiota

Food is a key factor influencing animal energy metabolism and gut microbiota. Bears exhibit remarkable dietary diversity, with distinct species showing preferences for specific food types. In Beijing Zoo, pandas primarily consume bamboo, bamboo shoots, corn, and fruits, with crude fiber constituting over half of their diet. Brown bears and black bears share a similar diet dominated by corn, fruits, and meat, with carbohydrates accounting for more than 70% of their intake. In contrast, polar bears rely predominantly on fish and meat, with protein comprising over 60% of their diet (Table S2).

**Figure 1:**
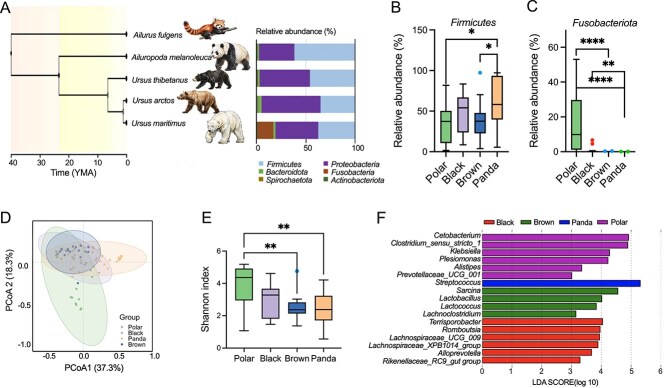
Differences in gut microbiota among four bear species. (A) The relative abundance of phyla in four species of bear fecal samples. (B,C) Significant differences in relative abundance of the *Fimicutes* and *Fusobacteriota* (Kruskal–Wallis test). (D) Principal coordinate analysis of Bray–Curtis dissimilarity of gut community compositions of four species of bear groups (ANOSIM). (E) Shannon index of four species of bear groups (Kruskal–Wallis test). (F) LEfSe analysis of relative abundance of the genus with significant differences in the four bear groups (LDA score > 3 and a significance of a < 0.05 are shown). Polar: polar bear, Black: Asian black bear, Panda: giant panda, Brown: brown bear. Data are means ± SEM. ^*^P < .05, ^**^P < .01, ^***^P < .001, ^****^P < .0001.

The composition of bear gut microbiota aligns partially with their evolutionary relationships (Fig. 1A), though environmental factors and dietary habits also play significant roles. We tested a total of 81 samples, and the clean tags for each sample sequencing are shown in Table S4. Profiling of microbial communities via 16S rRNA gene sequencing revealed higher alpha diversity in polar bears and black bears compared to pandas and brown bears (Shannon, *P* = .002, Fig. 1E). PCoA based on Bray–Curtis distances demonstrated significant differences in microbial community structure among the four species (ANOSIM, *P* = .001, *R*^2^ = 0.25, Table S5, Fig. 1D). At the phylum level, *Firmicutes* and *Proteobacteria* were predominant across all bears, whereas polar bears exhibited high abundances of *Fusobacteria* (Fig. S1A). *Firmicutes* levels in giant pandas significantly exceeded those in brown bears and polar bears (*P* = .0087, Fig. 1B), whereas *Fusobacteria* in polar bears were markedly elevated compared to other species (*P* < .0001, Fig. 1C). LEfSe identified distinct microbial biomarkers, *Lachnospiraceae_*XPB1014_group*, Romboutsia, Rikenellaceae_*RC9_gut_group, *Lachnospiraceae_*UCG_009, *Alloprevotella*, and *Terrisporobacter* dominated black bears, *Sarcina, Lachnoclostridium, Lactobacillus*, and *Lactococcus* were enriched in brown bears, *Klebsiella, Cetobacterium, Plesiomonas, Clostridium_*sensu_stricto_1*, Alistipes,* and *Prevotellaceae_*UCG_001 characterized polar bears. Giant pandas showed a predominance of *Streptococcus* (Fig. 1F). KEGG pathway enrichment analysis indicated that polar bears in winter exhibited heightened activity in metabolic pathways, including amino acid metabolism, energy metabolism, vitamin metabolism, and endocrine system functions, compared to the other three species, whereas Pandas exhibited heightened activity in nucleotide metabolism, xenobiotics biodegradation, metabolism of terpenoids and polyketides, and cell growth and death. (Supplementary Fig. S1B).

### Seasonal dynamics in bear gut microbiota

**Figure 2:**
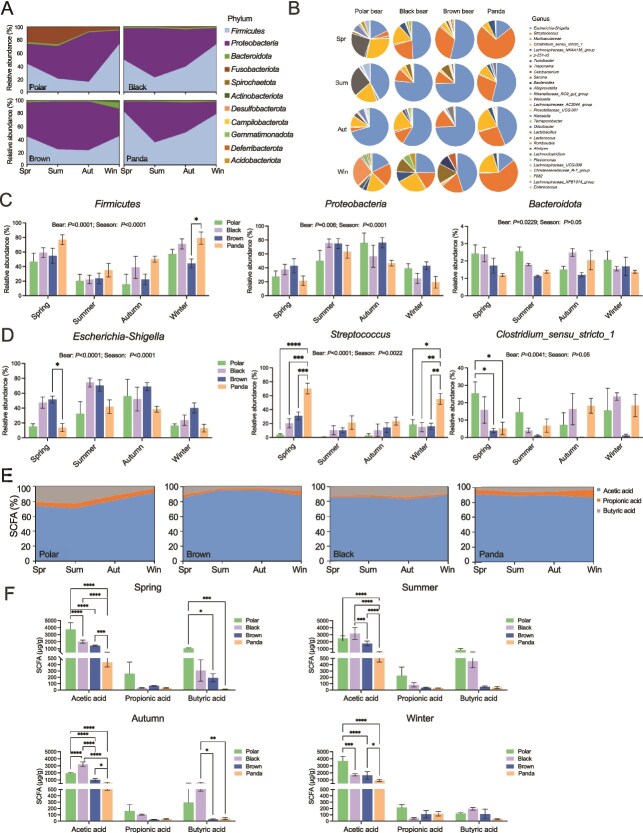
Differences in gut microbiota among four bear species in four seasons. (A) The relative abundance of phyla in four species of bear fecal samples in spring, summer, autumn and winter. (B) The relative abundance of genus in four species of bear fecal samples in spring, summer, autumn and winter. (C) Comparison of differences in four bears and seasons among the three phyla with the highest relative abundance (two-way ANOVA). (D) Comparison of differences in four bears and seasons among the three genus with the highest relative abundance (two-way ANOVA). (E) The proportions of acetic acid, propionic acid, and butyric acid in the feces of four species of bears. (F) Comparison of differences in four bears and seasons among the acetic acid, propionic acid, and butyric acid (one-way ANOVA). Polar: polar bear, Black: Asian black bear, Panda: giant panda, Brown: brown bear. Data are means ± SEM. Two-way ANOVA, with Tukey’s multiple comparisons test. ^*^P < .05, ^**^P < .01, ^***^P < .001, ^****^P < .0001.

The seasonal dynamics of gut microbiota in bears are illustrated in Fig. 2A. From spring to winter, the relative abundance of *Proteobacteria* in all four species exhibited a U-shaped trend, reaching its lowest level in summer and peaking in winter. Conversely, *Firmicutes* followed an inverse pattern, with the highest proportions observed in summer and autumn, and the lowest in spring and winter. In winter, *Firmicutes* was higher in Panda than black bear (*P* = .017, Fig. 2C). In spring, *Bacteroidota* was higher in polar bear than Panda (*P* = .045, Fig. 2C). Polar bears displayed elevated *Fusobacteriota* levels during spring and summer compared to other seasons. From a genus level, the impact of seasons indicates, in winter, polar bears are mainly dominated by *Weissella, Escherichia_Shigella*, *Clostridium_*sensu_stricto_1, and *Streptococcus*, whereas black bears also had *Turicibacter* and *Sarcina* in addition to *Escherichia_Shigella*, *Clostridium_*sensu_stricto_1, and *Streptococcus*, brown bears had the highest proportion of *Escherichia_Shigella*, as well as *Bacteroides* and *Treponema*. Pandas had the highest proportion of *Streptococcus*, followed by *Clostridium_*sensu_stricto_1*,* and *Escherichia_Shigella*. In spring, polar bears had a large proportion of *Clostridium_*sensu_stricto_1 more than half of Panda and brown bears (*P* < .05, Fig. 2D). The bacterial community of pandas was mainly composed of *Streptococcus* (*P* < .001, Fig. 2D). In summer, the intestines of the four bear species are mainly composed of *Escherichia_Shigella*, The trends in autumn and summer were similar (Fig. 2B). Comparison results of phyla and genus pairwise of four bears in four seasons were in Tables S6, S7 (Mann–Whitney *U* test, *P* < .05).

We tested the fecal short chain fatty acid content of four types of bears in four seasons (Fig. 2E). Acetic acid accounts for over 80%, followed by propionic acid and butyric acid. The proportion of butyric acid in polar bears increases in spring and summer, whereas in pandas it increases in summer. In brown bears, the proportion of butyric acid decreases in summer and autumn (Fig. 2F). In spring, polar bears had significantly higher levels of acetic acid than black bears (*P* < .0001), brown bears (*P* < .0001), and pandas (*P* < .0001). Butyric acid is significantly higher than that of brown bears (*P* = .0215) and pandas (*P* = .0008). In summer, the acetic acid levels of polar bears (*P* < .0001), brown bears (*P* < .0001), and black bears (*P* < .0001) were significantly higher than pandas. The butyric acid content of black bears in autumn is significantly higher than that of brown bears (*P* = .0118) and pandas (*P* = .0039). In winter, polar bears had significantly higher levels of acetic acid than black bears (*P* = .0006), brown bears (*P* < .0001), and giant pandas (*P* < .0001).

To explore the functional potential of the gut microbiota across different bear species, we combined 16S rRNA sequencing data with the KEGG database for functional prediction. The enrichment analysis revealed significant functional differences among the gut microbiota of the four bear species. There were significant functional differences among the four bear gut microbiota. In winter, the main functions of polar bears are lipid metabolism and nucleotide metabolism, The function of the giant panda microbiota was concentrated in translation and cell growth. The brown bear microbiota was mainly used for metabolism and cellular processes, whereas the black bear microbiota functions mainly in the biological enzyme pathway (Fig. S2A). In summer, The functional differences of the four bear gut microbiota are relatively small. The main functions of polar bears are energy metabolism, cofactors and vitamins metabolism, sensory and endocrine system, and cell communication, whereas the giant panda microbiota functions mainly in the membrane transport (Fig. S2B).

### Transplantation of winter bear gut microbiota alters host metabolic phenotypes and gut microbiota in mice

**Figure 3:**
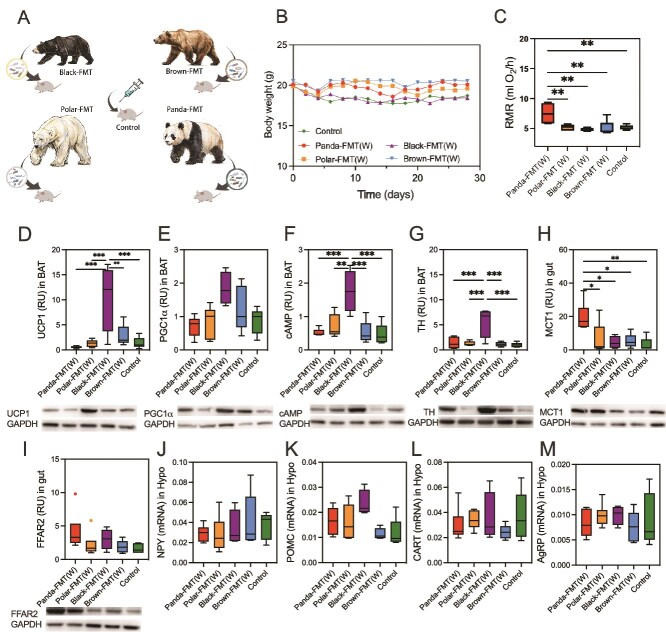
Winter bear fecal microbiota transplant alters metabolic phenotypes, neurotransmitters. (A) Experimental design of FMT. (B) Body weight of five FMT groups (repeated measures ANOVA). (C) RMR of five FMT groups (one-way ANOVA). (D) Relative expression of UCP1 in BAT (one-way ANOVA). (E) Relative expression of PGC1-α in BAT (one-way ANOVA). (F) Relative expression of cAMP in BAT (one-way ANOVA). (G) Relative expression of TH in BAT (one-way ANOVA). (H) Relative expression of MCT1 in small gut (one-way ANOVA). (I) Relative expression of FFAR2 in small gut (one-way ANOVA). (J) Relative expression of NPY in hypothalamus (one-way ANOVA). (K) Relative expression of POMC in hypothalamus (one-way ANOVA). (L) Relative expression of CART in hypothalamus (one-way ANOVA). (M) Relative expression of AgRP in hypothalamus (one-way ANOVA). Control, mice with saline gavage, Polar-FMT (W), mice with winter polar bear fecal microbiota gavage, Panda-FMT (W), mice with winter panda fecal microbiota gavage, Brown-FMT (W), mice with winter brown bear fecal microbiota gavage, Black-FMT (W), mice with winter black bear fecal microbiota gavage. Data are means ± SEM, Kruskal–Wallis test, ^*^P < .05, ^**^P < .01, ^***^P < .001, ^****^P < .0001.

To investigate the functional impact of winter bear gut microbiota, we performed FMT from four bear species into mice (Fig. 3A). whereas no significant body weight changes were observed among groups (*P* > .05, Fig. 3B). However, the rest metabolic rate of the Panda-FMT group was significantly higher than that of other groups (*P* = .0008, Fig. 3C). Due to the transplanted winter microbiota, we are more concerned about the energy metabolism and heat production of mice. The expression levels of UCP1 (*P* = .0002) and cAMP (*P* = .0001) in the BAT of the Black-FMT group were significantly higher than those of the other four groups (Fig. 3D–F). TH expression in BAT increased in the Black-FMT group compared to that of the other groups (*P* = .0001, Fig. 3G). The receptor involved in the transportation of substances such as short chain fatty acids in the intestine were detected, and we found that there was no significant difference among the groups of FFAR2 (*P* = .166, Fig. 3I). Monocarboxylate transporter (MCT1) was significantly higher in the Panda-FMT group than in other groups (*P* = .0026, Fig. 3H). We detected neurons related to food intake in the hypothalamus, and although there were no significant differences, it was still found that in the Black-FMT and Brown-FMT groups, the promoting food intake neurons NPY was higher, whereas the POMC inhibiting food intake was lower (*P* > .05, Fig. 3J–M).

**Figure 4:**
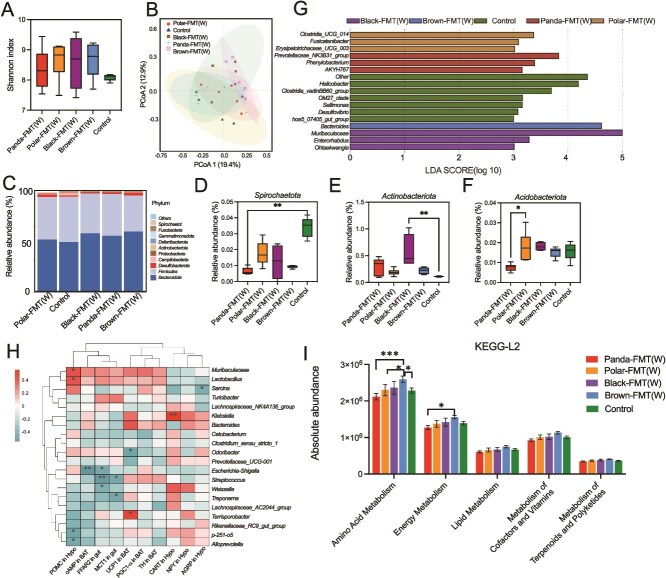
Winter bear fecal microbiota transplant alters bacterial diversity and composition. (A) Shannon index of five FMT groups (Kruskal-Wallis test). (B) Principal coordinate analysis of Bray–Curtis dissimilarity of gut community compositions of five groups. Group differences were tested by ANOSIM. (C) The relative abundance of phyla in five groups. (D–F) Relative abundance of the Spirochaetota, Actionbacteriota and Acidobacteriota (Kruskal–Wallis test). (G) LDA scores of the differentially abundant taxa enriched in microbiota from five groups (taxa with LDA score > 3 and a significance of a < 0.05 are shown). (H) Heatmap showing the correlation between specific genus and physiological measurements (Spearman). (I) Functional prediction results of KEGG based on 16S rRNA sequence. Control, mice with saline gavage, Polar-FMT (W), mice with winter polar bear fecal microbiota gavage, Panda-FMT (W), mice with winter panda fecal microbiota gavage, Brown-FMT (W), mice with winter brown bear fecal microbiota gavage, Black-FMT (W), mice with winter black bear fecal microbiota gavage. Data are means ± SEM. Kruskal-Wallis test, ^*^P < .05, ^**^P < .01, ^***^P < .001, ^****^P < .0001.

After winter gut microbiota transplantation, there was no significant difference in alpha diversity of gut microbiota among the five groups (*P* > .05, Fig. 4A), whereas PCoA based on Bray-Curtis distance showed significant differences in microbial community structure among the five groups (ANOSIM, *P* = .004, *R*^2^ = 0.26, Fig. 4B, Table S8). The *Spirochaetota* of Panda-FMT group was significantly lower than control group (*P* = .0024, Fig. 4D), the *Actionbacteriota* of Black-FMT group was significantly higher than control (*P* = .0088, Fig. 4E), whereas the *Acidobacteriota* of Panda-FMT group was significantly lower than Polar-FMT group (*P* = .0073, Fig. 4F). LEfSe analysis showed that *Bacteroides* predominated in Brown-FMT mice, *Prevotellaceae_*NK3B31_group*, Phenylobacterium* and AKYH767 were predominated in Panda-FMT, Clostridia_UCG_014, *Fusicatenibacter* and *Erysipelotrichaceae_UCG_003* predominated in Polar-FMT mice, *Muribaculaceae*, *Enterorhabdus*, and *Ohtaekwangia* were the most abundant bacterium in the intestines of Black-FMT mice (Fig. 4G). The heatmap showed the correlation between specific genera and physiological measurements (Fig. 4H). The genera of *Terrisporobacter* was positive correlated with UCP1 expression in BAT. *Klebsiella* was positive correlated with CART in hypothalamus. *Muribaculaceae* and *Lactobacillus* were positive correlated with POMC in hypothalamus. The KEGG enrichment pathway results showed that, amino acid metabolism in Brown-FMT group was higher than Panda-FMT (*P* < .0001) and Polar-FMT (*P* = .0228) and control group (*P* = .0215), energy_metabolism in Brown-FMT group was higher than Panda-FMT group (*P* = .0305, Fig. 4I).

### Transplantation of summer bear gut microbiota alters host metabolic phenotypes and gut microbiota in mice

**Figure 5:**
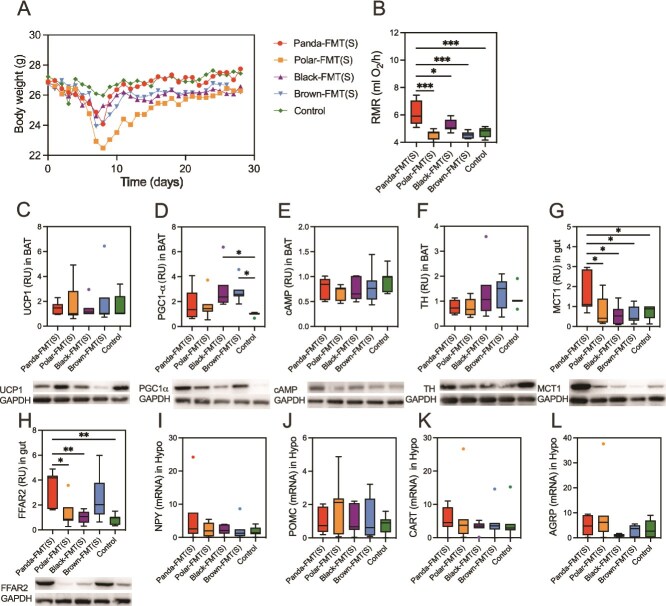
Summer bear fecal microbiota transplant alters metabolic phenotypes, neurotransmitters. (A) Body weight of five FMT groups (repeated measures ANOVA). (B) RMR of five FMT groups (one-way ANOVA). (C) Relative expression of UCP1 in BAT (one-way ANOVA). (D) Relative expression of PGC1-α in BAT (one-way ANOVA). (E) Relative expression of cAMP in BAT (one-way ANOVA). (F) Relative expression of TH in BAT (one-way ANOVA). (G) Relative expression of MCT1 in small gut (one-way ANOVA). (H) Relative expression of FFAR2 in small gut (one-way ANOVA). (I) Relative expression of NPY in hypothalamus. (J) Relative expression of POMC in hypothalamus (one-way ANOVA). (K) Relative expression of CART in hypothalamus (one-way ANOVA). (L) Relative expression of AgRP in hypothalamus (one-way ANOVA). Control, mice with saline gavage, Polar-FMT (S), mice with summer polar bear fecal microbiota gavage, Panda-FMT (S), mice with summer panda fecal microbiota gavage, Brown-FMT (S), mice with summer brown bear fecal microbiota gavage, Black-FMT (S), mice with summer black bear fecal microbiota gavage. Data are means ± SEM, Kruskal–Wallis test, ^*^P < .05, ^**^P < .01, ^***^P < .001, ^****^P < .0001.

To investigate the functional impact of summer bear gut microbiota, we performed FMT from four bear species into mice. The body weight of mice showed no significant changes after bacterial community transplantation (Fig. 5A), although the Panda-FMT group exhibited a slightly higher trend. However, the RMR of the Panda-FMT group was significantly higher than that of the other groups (*P* < .001, Fig. 5B). In BAT, the expression levels of PGC1-α (*P* = .011, Fig. 5D) in the black bear-FMT and Brown-FMT groups were significantly elevated compared to the control group, whereas no significant differences were observed in UCP1, cAMP, or TH expression, suggesting that the summer microbiota had limited effects on thermogenesis (*P* > .05, Fig. 5C–F). The SCFA receptors MCT1 (*P* = .0091) and FFAR2 (*P* = .0009) were upregulated in the Panda-FMT group (Fig. 5G, H). Although we analyzed hypothalamic neurons associated with food intake regulation, no significant differences were found (*P* > .05, Fig. 5I–L).

**Figure 6:**
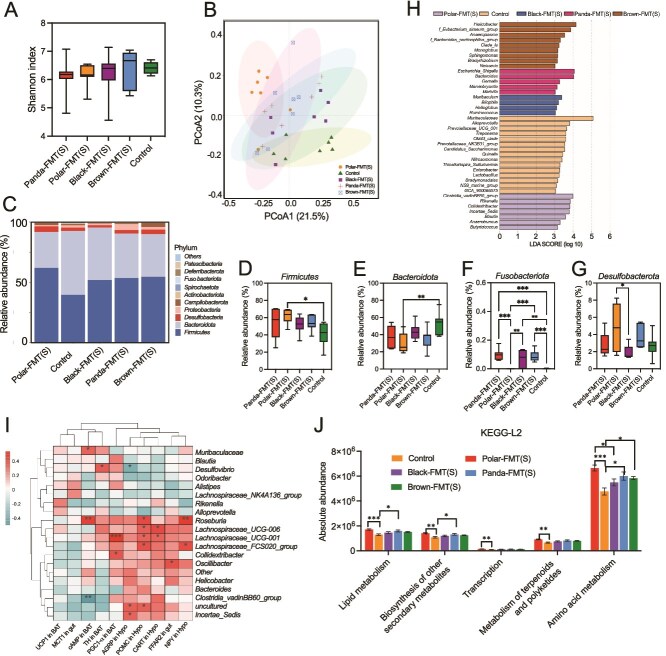
Summer bear fecal microbiota transplant alters bacterial diversity and composition. (A) Shannon index of five FMT groups (Kruskal-Wallis test). (B) Principal coordinate analysis of Bray–Curtis dissimilarity of gut community compositions of five groups. Group differences were tested by ANOSIM. (C) The relative abundance of phyla in five groups. (D–G) Relative abundance of the Fimicutes, Bacteroidota, Fusobacteriota, and Desulfobacteriota (Kruskal–Wallis test). (H) LDA scores of the differentially abundant taxa enriched in microbiota from five groups (taxa with LDA score > 3 and a significance of a < 0.05 are shown). (I) Heatmap showing the correlation between specific genus and physiological measurements (Spearman). (J) Functional prediction results of KEGG based on 16S rRNA sequence (Kruskal–Wallis test). Control, mice with saline gavage, Polar-FMT (S), mice with summer polar bear fecal microbiota gavage, Panda-FMT (S), mice with summer panda fecal microbiota gavage, Brown-FMT (S), mice with summer brown bear fecal microbiota gavage, Black-FMT (S), mice with summer black bear fecal microbiota gavage. Data are means ± SEM, Kruskal–Wallis test, ^*^P < .05, ^**^P < .01, ^***^P < .001, ^****^P < .0001.

After transplantation of the summer gut microbiota, the Shannon index showed no significant differences among the five groups (Fig. 6A). In contrast, PCoA based on Bray–Curtis distances revealed significant structural differences in microbial communities across groups (ANOSIM, *P* = .001, *R*^2^ = 0.31, Fig. 6B, Table S9). The *Firmicutes*/*Bacteroidota* ratio in the Polar-FMT group was significantly higher than that of the control group (Fig. 6C). The *Firmicutes* of Polar-FMT group was significantly higher than control group (*P* = .0294, Fig. 6D), the *Bacteroidota* was significantly lower than control (*P* = .0119, Fig. 6E). *Desulfobacterota* of Polar-FMT group was significantly higher than Black-FMT group (*P* = .0297, Fig. 6G). *Fusobacteriota* showed significant differences among these groups (*P* < .0001, Fig. 6F). LEfSe analysis showed that *Helicobacter, Monoglobus, Sphingomonas, Bradyrhizobium,* and *Neisseria* were predominated in Brown-FMT mice, *Rikenella, Butyricicoccus, Anaerotruncus,* and *Blautia* were predominated in Polar-FMT, *Bacteroides*, *Gemella, Marvinbryantia*, and *Marivita* predominated in Panda-FMT mice, *Muribaculum, Bilophila, Halioglobus,* and *Ruminococcus* were the most abundant bacterium in the intestines of Black-FMT mice (Fig. 6H). The heatmap showed the correlation between specific genera and physiological measurements (Spearman, Fig. 6I). The genera of *Colidextribacter* and *Lachnospiraceae_*UCG_001 were positive correlated with PGC1α expression in BAT. *Roseburia*, *Lachnospiraceae_*FCS020_group, *Lachnospiraceae_*UCG_001, and *Lachnospiraceae*_UCG_006 were positive correlated with POMC in hypothalamus. *Roseburia*, and *Lachnospiraceae*_FCS020_group were positive correlated with NPY expression in hypothalamus. The KEGG enrichment pathway results showed that, amino acid metabolism in Polar-FMT group was higher than Black-FMT and control group (*P* < .0001), lipid metabolism and biosynthesis of other secondary metabolites in Polar-FMT (*P* < .05) and Panda-FMTgroup (*P* < .05) were higher than control group, transcription and metabolism of terpenoids and polyketides in Polar-FMT group were higher than control group (*P* < .05, Fig. 6J).

**Figure 7:**
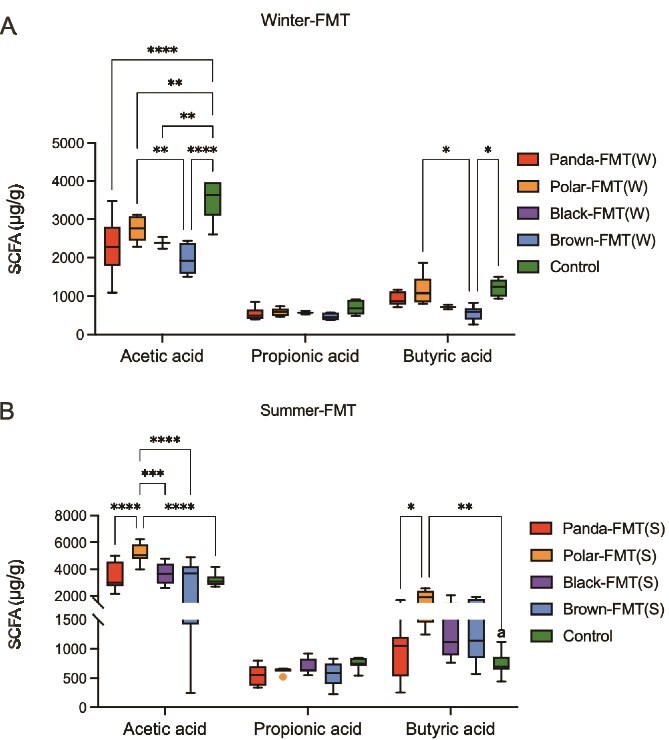
Bear fecal microbiota transplant alters content of acetic acid, propionic acid, and butyric acid. （A）Winter fecal microbiota transplant alters content of acetic acid, propionic acid, and butyric acid (one-way ANOVA). (B) Summer fecal microbiota transplant alters content of acetic acid, propionic acid, and butyric acid (one-way ANOVA). Control, mice with saline gavage, Polar-FMT (W), mice with winter polar bear fecal microbiota gavage, Panda-FMT (W), mice with winter panda fecal microbiota gavage, Brown-FMT (W), mice with winter brown bear fecal microbiota gavage, Black-FMT (W), mice with winter black bear fecal microbiota gavage. Polar-FMT (S), mice with summer polar bear fecal microbiota gavage, Panda-FMT (S), mice with summer panda fecal microbiota gavage, Brown-FMT (S), mice with summer brown bear fecal microbiota gavage, Black-FMT (S), mice with summer black bear fecal microbiota gavage. Data are means ± SEM, Kruskal–Wallis test, ^*^P < .05, ^**^P < .01, ^***^P < .001, ^****^P < .0001.

We measured the content of key metabolites after microbiota transplantation, including acetic acid, propionic acid, and butyric acid. Winter and summer transplant groups exhibit different patterns. In the winter transplantation group, the acetic acid content was lower of the four bear-FMT groups than that of the control group (*P* < .0001, Fig. 7A), and the acetic acid content in Polar-FMT was higher than that in Brown-FMT group (*P* = .0022, Fig. 7A), there was no difference in propionic acid, but the content of butyric acid in Polar-FMT group was higher than that in Brown-FMT group (*P* = .0311, Fig. 7A). In the summer transplantation group, the acetic acid content was higher of the Polar-FMT group than others (*P* < .0001, Fig. 7B), there was no difference in propionic acid, but the content of butyric acid in Polar-FMT group was higher than that in Panda-FMT and control groups (*P* = .0374, Fig. 7B).

### Regulation of gene expression in recipient mice after FMT

**Figure 8:**
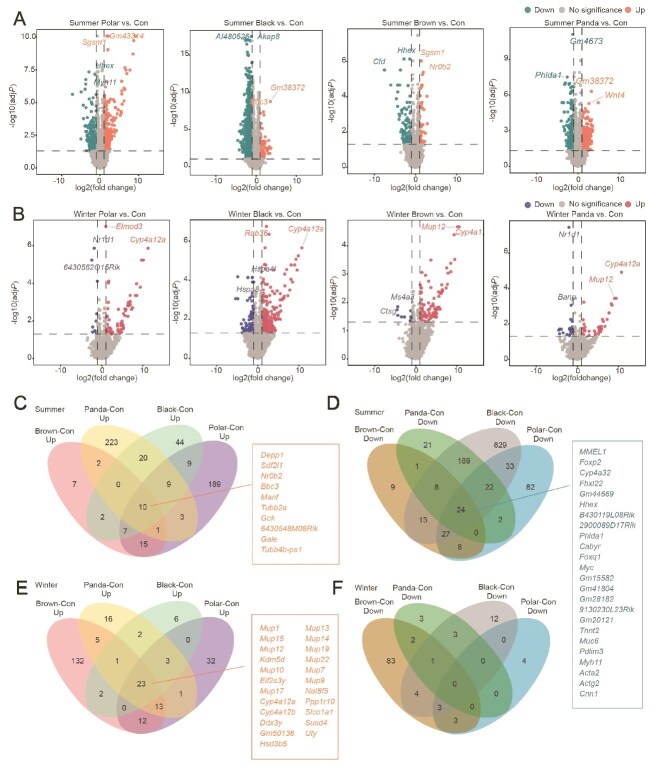
Volcano plots of the DEGs and Venn plots of DEGs. (A) Volcano plots of upregulated and downregulated DEGs in summer gut microbiota transplantation. (B) Volcano plots of upregulated and downregulated DEGs in winter gut microbiota transplantation. (C–F) The Venn diagrams showed the number and names of DEGs between the different groups.

Transcriptome sequencing of mouse liver revealed distinct gene expression patterns between FMT and control groups. Volcano plots illustrated the impact of donor bear microbiota on hepatic gene expression of mice, with clear separation between winter and summer transplantation groups (Fig. 8A, B). In the summer gut microbiota transplantation cohort, 10 upregulated DEGs and 24 downregulated DEGs were shared across all four FMT groups (Figs. 8C, D). Conversely, 23 upregulated DEGs were commonly identified in the four FMT groups receiving winter gut microbiota (Figs. 8E, F). The genes upregulated in the winter gut microbiota transplantation group were mainly: *Mup1 15 12 10 13 14 19 22 7 9*, *kdm5d*, *nat8f5*, *Ppp1r10*, *Cyp4a12ab*, *Hsd3b5*, *Susd4*, *Ddx3y*, *Gm50136*, *Slco1a1*, and *Uty*. The genes upregulated in the summer gut microbiota transplantation group were mainly: *Depp1*, *Sdf2l1*, *Nr0b2*, *Bbc3*, *Manf*, *Tubb2a*, *Gck*, *Gale*, and *Tubb4b-ps1*. The genes downregulated in the summer gut microbiota transplantation group were mainly: *MMEL1*, *Foxp2*, *Cyp4a32*, *Fbxl22*, *Hhex*, *B430119L08Rik*, *Phlda1*, *Cabyr*, *Foxq1*, *Myc*, *Muc6*, *Tnnt2*, *Pdlim3*, *Myh11*, *Acta2*, and *Cnn1*.

## Discussion

### Convergent evolution of gut microbiota is a universal strategy for bears energy challenges

The bear family includes eight species, ranging in diet from strict herbivorous (giant panda), omnivorous (Malay bear, etc.) to strict carnivorous (polar bear) [19], their gut microbial communities reflect adaptations to their dietary ecology and digestive physiology. A phylogenetic analysis using red pandas as a reference shows that polar bears and brown bears had the closest genetic relationship, followed by black bears, whereas giant pandas had the farthest relationship with the other three types of bears. In our study, the gut microbiota of the four types bears was mainly composed of *Firmicutes* and *Proteobacteria*, which is consistent with other studies [4, 8, 20, 21]. At the genus level, it is mainly occupied by *Escherichia-Shigella*, *Streptococcus*, and *Clostridium_ sensu_stricto_1*. Despite analyzing captive individuals, our results demonstrate a phylogenetic signal in gut microbiota structure: black bears and Pandas clustered closely, whereas polar bears and brown bears exhibited higher microbial similarity. This aligns with broader comparative studies, for instance, a meta-analysis of 18 wild non-human primate species revealed that host phylogeny exerts a stronger influence on gut microbiota composition and function than dietary niche [22]. Gaulke et al. conducted 16S rRNA analysis on feces from 32 species of 10 mammalian orders, and the results showed that the correlation between host feeding strategies (carnivorous, omnivorous, herbivorous) and host gut physiology (simple gut, post fermentation gut, pre fermentation gut) and these beta-diversity indicators was weak, and the degree of influence was lower than that of host genes [23]. The consistency between host and microbial phylogenetics has been confirmed in numerous studies [24–29]. Although captive black bears and brown bears shared comparable diets, their microbiota diverged significantly, further underscoring the primacy of evolutionary history over dietary inputs. Specifically, black bears enriched *Lachnospiraceae*, whereas brown bears showed dominance of *Lactobacillus* and *Lactococcus*.

Diet plays a pivotal role in shaping gut microbiota. For instance, giant pandas, whose diet consists of bamboo-derived fiber, exhibited the highest fecal abundance of *Clostridium* species [30]. Our study further revealed that the relative abundance of *Firmicutes* in panda feces was significantly higher than that in polar bears and brown bears. Specifically, *Streptococcus* dominated the fecal microbiota of pandas, consistent with their fiber-rich diet. In contrast, black bears and brown bears consuming carbohydrate-dominated diets showed fecal microbiota enriched with *Escherichia-Shigella* and *Streptococcus*. Polar bears, as hypercarnivores, displayed a distinct microbial profile characterized by *Escherichia-Shigella, Clostridium_*sensu_stricto_*1*, and *Cetobacterium*, with *Fusobacteria* phylum abundance substantially exceeding that of other bear species. Alpha diversity of gut microbiota varied markedly across species, polar bears exhibited the highest diversity, whereas pandas showed the lowest, potentially reflecting the limited dietary complexity of bamboo specialists. This observation aligns with vertebrate studies demonstrating diet as the strongest predictor of gut microbial alpha diversity [31]. Beyond diet, the environmental factors and foraging ecology further modulate the gut microbiota of bear. For example, polar bears feeding on marine mammals and fish likely harbor microbiota adapted to high-fat/protein metabolism, pandas enriched *Clostridium butyricum*, a keystone species enhancing bamboo fiber degradation [30], black bears and brown bears exhibited higher abundances of *Lachnospiraceae* and *Lactobacillus*, which facilitated carbohydrate metabolism (e.g. pectin digestion in fruits). Functional analyses corroborated these patterns, linking microbiota profiles to metabolic pathways such as glycolysis and fatty acid oxidation.

### Despite a specialized diet, panda gut microbiota evolution still follows the bear blueprint

Although captive bears live in stable environment, their gut microbiota still exhibits significant seasonal fluctuations. This study found that four species of Ursidae exhibit consistent microbial changes at the phylum level: *Firmicutes* abundance peaks in winter and spring, but decreases in summer and autumn, whereas *Proteobacteria* shows the opposite trend. This seasonal restructuring may achieve energy regulation through the short chain fatty acids metabolic pathway, hibernating brown bears and Polar active polar bears require thick walled bacteria mediated SCFAs (such as acetic acid, propionic acid, butyric acid) to enhance heat production efficiency [32]. It is worth noting that the concentration of acetic acid in polar bear feces was higher in winter than in summer, whereas the content of butyric acid in brown bear feces significantly increases in winter and spring. Experimental evidence has shown that both types of SCFAs can enhance non shivering thermogenesis in BAT by activating the AMPK signaling pathway [33, 34].

Anatomical studies had shown that bear animals retain typical carnivorous digestive tract features， including short small intestine, degenerated hindgut, and lack of cecum [35, 36]. Similar to other herbivorous mammals that rely on microbial degradation of cellulose, the Ursidae family relies entirely on gut microbiota to convert plant fiber into bioavailable nutrients [37, 38]. Taking the giant panda, which exclusively feeds on bamboo, as an example, its gut microbiota system is still homologous to that of the bear family, but bamboo fiber degradation is achieved through the enrichment of cellulase active bacteria such as *Clostridium* [8, 39]. Similarly, the increased abundance of *Fusobacteria* in polar bears during spring and summer may be related to their periodic feeding of fish, which can assist in energy extraction from high protein and high-fat foods [40]. The genus *Clostridium*, which has carbohydrate hydrolysis function, is not only found in the intestines of pandas, but also distributed in captive black bears and polar bears, suggesting that the bear family may share a universal microbial metabolism toolbox, achieving dietary adaptation through changes in microbial community composition rather than unique strains.

The significant enrichment of *Streptococcus* in pandas reveals their unique nutritional strategy, *Streptococcus* compensates for its weakened carbohydrate metabolism ability compared to omnivores by enhancing the expression of amino acid metabolism genes [41]. This modular adaptation mode of microbial community function is also reflected in other specialized feeding bear species such as Andean bears [20]. Research has confirmed that although different bear species develop unique microbial functional characteristics due to feeding habits, their core microorganisms always maintain a buffer system to cope with environmental pressures, and maintain host energy homeostasis through the SCFAs metabolic network. This discovery demonstrates that the dietary specialization of pandas has not broken through the common adaptation framework of bear family, but has achieved ecological niche expansion under anatomical limitations through microbial mediated metabolic plasticity.

### Bear gut microbiota reprograms host energy allocation in mice

To further investigate the role of bear gut microbiota in energy metabolism, this study transplanted gut microbiota from bears collected during winter and summer into mice and measured relevant energy metabolism indicators. Specifically, mice transplanted with winter black bear microbiota showed a significant increase in the expression of UCP1 (uncoupling protein 1) and cAMP in BAT, indicating enhanced thermogenic capacity. UCP1 is a key protein in BAT responsible for non-shivering thermogenesis, and its increased expression suggests that mice can more efficiently convert energy into heat to cope with cold environments [42]. Additionally, the expression of FFAR2 (free fatty acid receptor 2) was also significantly increased in these mice, suggesting that their gut microbiota may influence host energy metabolism by modulating SCFAs [43]. Through 16S rRNA gene sequencing and co-expression analysis, it was found that *Enterococcus, Terrisporobacter* and *Bacteroides* were positive correlated with UCP1 expression in BAT, indicating their potential role in thermogenesis. Similarly, correlations between specific genera and hypothalamic CART or FFAR2 expression suggest their involvement in appetite regulation and SCFA sensing. In contrast, mice transplanted with summer bear microbiota exhibited minimal changes in energy metabolism, particularly no significant differences in the expression of thermogenic genes. This indicates that bear gut microbiota in winter may had stronger functional capabilities on regulating host energy metabolism, especially in response to cold environments. In addition to energy metabolism, this study also explored the impact of gut microbiota on host appetite regulation. By measuring the expression of appetite-related neuropeptides in the mouse hypothalamus (such as NPY, AgRP, POMC, and CART), it was found that mice transplanted with winter bear microbiota showed certain changes in appetite regulation. Specifically, mice transplanted with winter black bear microbiota and winter brown bear microbiota exhibited increased expression of the orexigenic neuropeptide NPY (neuropeptide Y) and decreased expression of the anorexigenic neuropeptide POMC (pro-opiomelanocortin). This suggests that winter bear gut microbiota might regulate host appetite to increase energy intake in response to elevated energy demands in cold environments [44]. Furthermore, certain gut microbiota genera (such as *Enterococcus, Treponema*, and *Bacteroides*) were found to be positively correlated with the expression of UCP1 and CART, indicating their potential roles in regulating both energy metabolism and appetite. These findings further support the critical role of gut microbiota in host energy homeostasis, suggesting that they may bidirectionally regulate energy intake and expenditure through the gut-brain axis.

### KEGG pathway analysis highlights metabolic function differences

KEGG pathway analysis demonstrated distinct metabolic functional profiles across FMT groups. In winter-transplanted cohorts, the brown bear-FMT group exhibited elevated amino acid metabolism compared to giant panda-FMT, polar bear-FMT, and control groups, with concurrent enhancement in energy metabolism relative to giant pandas. This metabolic signature may reflect brown bears’ hibernation adaptation, where protein catabolism sustains basal physiological functions and mitigates starvation stress during prolonged torpor [4]. Although hibernation reduces overall energy expenditure, pre-hibernation hyperphagia drives fat accumulation and transient metabolic surges to meet seasonal demands [45]. In contrast, polar bears-non-hibernating Arctic specialists-prioritize lipid metabolism to exploit their high-fat marine mammal diet, minimizing amino acid reliance [46]. Giant pandas displayed suppressed winter energy metabolism consistent with their nutrient-poor bamboo diet. Field metabolic rate (FMR) studies confirm their exceptionally low energy expenditure compared to ursid, compensated by winter metabolic upregulation for thermogenesis-an adaptation constrained by bamboo’s caloric limitations [47].

The summer microbiota transplantation experiment showed that the polar bear FMT group enhanced immune regulation and oxidative stress resistance by upregulating terpenoid/polyketide metabolic pathways, which may be a survival strategy to cope with food shortage and pathogen exposure in the Arctic summer. The giant panda FMT group enhances lipid metabolism and secondary metabolite synthesis pathways, improving the energy extraction efficiency of bamboo derived phenolic/flavonoid compounds and storing fat for the reproductive cycle [48]. This microbial-mediated metabolic plasticity enables multi-level adaptation from nutrient extraction to systemic physiological regulation.

### Microbial regulation of metabolic gene network

Transcriptomic analysis revealed that genes commonly upregulated in the winter transplantation group mainly participate in lipid metabolism. Some *Mups* genes may be involved in lipid metabolism and energy balance or possess antimicrobial or immunomodulatory functions [49, 50]. *Cyp4a12a/b* genes are involved in fatty acid oxidation, particularly catalyzing the metabolism of arachidonic acid and other long-chain fatty acids, generating bioactive lipid mediators [51]. *Hsd3b5* is involved in steroid hormone synthesis and metabolism [52].

In the summer microbiota transplantation group upregulated genes primarily regulate carbohydrate metabolism. *NR0B2* inhibits the activity of various nuclear receptors by forming heterodimers, thereby regulating bile acid synthesis, lipid metabolism, and glucose homeostasis [53]. *GCK* is an enzyme that catalyzes glucose phosphorylation, mainly expressed in the liver and pancreatic β-cells. It is a key glucose-sensing enzyme that detects blood glucose levels and regulates insulin secretion [54]. Among the downregulated genes in the summer group, *Cyp4a32* participates in fatty acid metabolism, specifically ω-hydroxylation of long-chain fatty acids, contributing to maintaining lipid balance [55].

This metabolic rhythm of winter lipid and summer carbon is universal among the four bear species. In winter, energy reserves are strengthened through lipid metabolism to cope with low temperature stress, whereas in summer, sugar metabolism is optimized to meet high energy consumption needs. The microbiota host interaction shapes a unified strategic framework for bear animals to cope with environmental fluctuations through the temporal regulation of metabolic gene networks.

### Summary

This study, by analyzing the gut microbiota from four species of bears and conducting transplantation experiments, has uncovered the crucial role of gut microbiota in the energy metabolism and appetite regulation of bears. The seasonal variations in gut microbiota demonstrate the adaptability of bears to environmental changes, particularly during periods of high energy demand like winter. These microbiota assist bears in regulating their energy metabolism and appetite to meet varying energy needs throughout the year. Our study highlights the co-evolution of bear gut microbiota with host dietary niches and provides mechanistic insights into microbial regulation of energy homeostasis. The gut microbiota of giant pandas does not exhibit uniqueness in regulating energy metabolism and seasonal adaptation, but rather reflects the plastic microbial strategy formed by the evolution of bears. This discovery challenges the traditional paradigm of defining species specificity based on nutritional adaptation, emphasizing are understanding of evolution from the perspective of microbial function. For the conservation of the bear family, maintaining the energy metabolism elasticity driven by microorganisms may be more ecologically significant than focusing on a single species.

Our research has many deficiency. Firstly, captive diets lack the diversity and unpredictability of wild foraging, potentially masking subtle host-microbiota adaptations. Then, captive bears experience no food scarcity, predation, or climate extremes, potentially dampening seasonal metabolic adaptations seen in wild populations. Secondly, compared to wild animals of the same species, antibiotics, cramped habitats, and human contact can all alter the composition of the microbial community. Therefore, further research is needed on the gut microbiota and adaptive evolution of wild animals in the future.

## Supplementary Material

Supplementary_wraf201

## Data Availability

16S rRNA sequence data are available in the NCBI Sequence Read Archive (SRA) under BioProject accession PRJNA1207124 and PRJNA1260068. And all the other data supporting the conclusions of this study is available in the paper and supplemental materials.
