# Supplementary material for: Convergent gut microbial functional strategies drive energy metabolism adaptation across Ursidae species and challenge the uniqueness of giant panda
Source: ISME J. 2025 Sep 3;19(1):wraf201. doi: 10.1093/ismejo/wraf201 (PMC12459253; doi:10.1093/ismejo/wraf201)
Supplement: Supplementary_wraf201 [file supplementary_wraf201.pdf]

Convergent gut microbial functional strategies drive energy metabolism adaptation across Ursidae species and challenge the uniqueness of giant panda

Supplementary figures

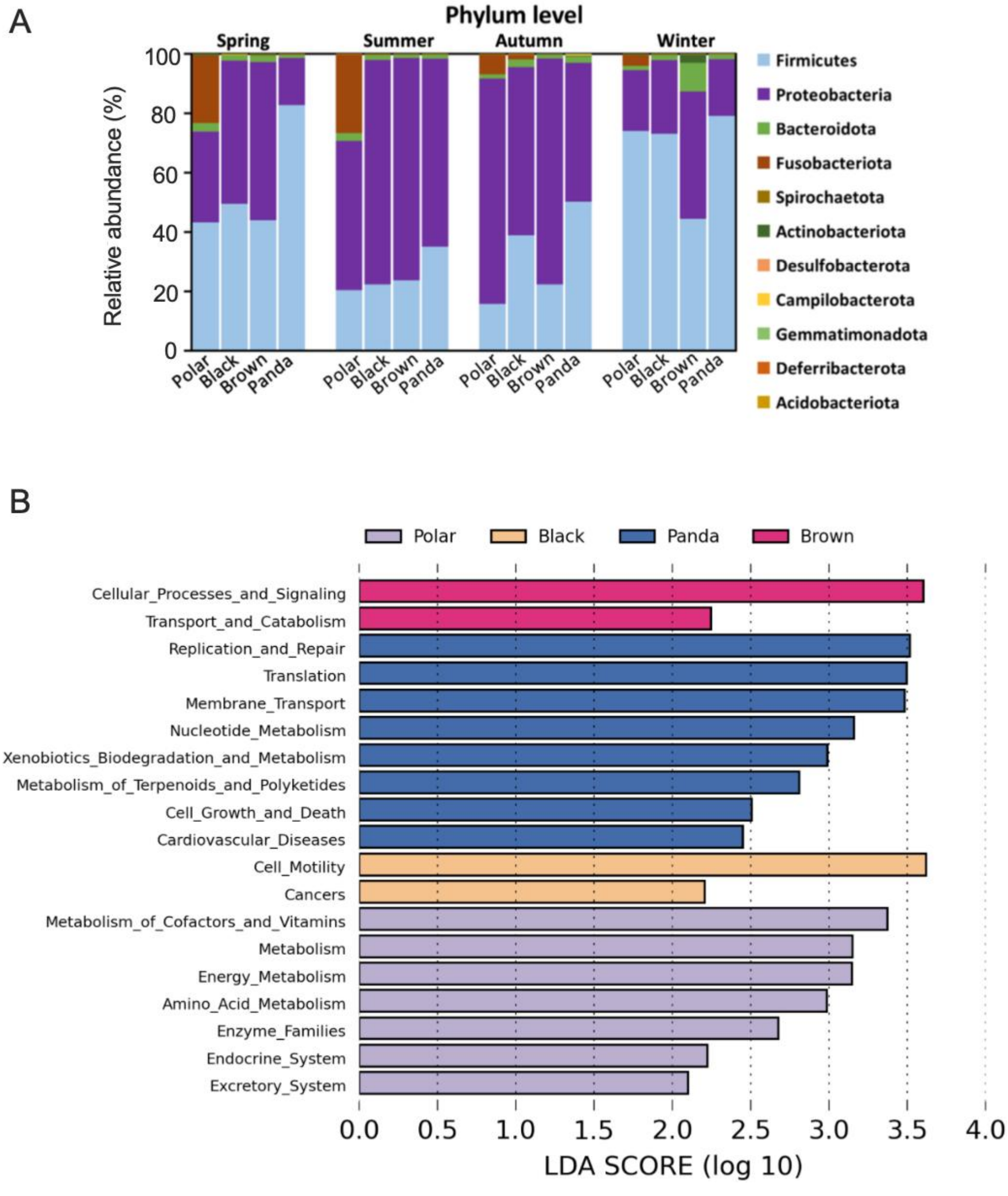

Figure S1. Differences in gut microbiota among four bear species.
(A) The relative abundance of phyla in four species of bear fecal samples.
(B) Functional prediction results of KEGG based on 16S rRNA sequence in four species of bear (LDA>2).

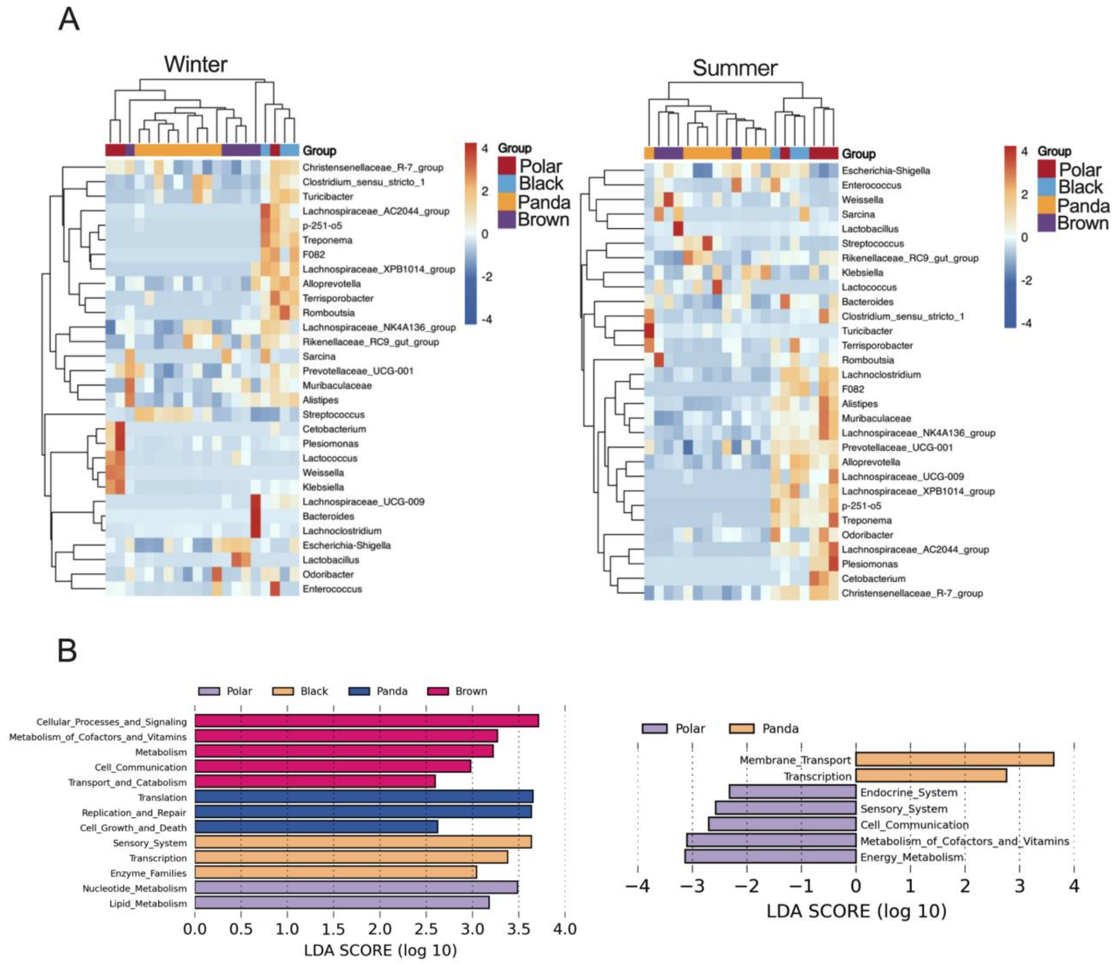

Figure S2 Comparison of microbial composition and function of four bear species in winter and summer.

(A) Cluster heatmap showing the proportions of amplicon sequence variants classified at the top 30 genus rank in winter and summer.

(B) Functional prediction results of KEGG based on 16S rRNA sequence in winter and summer (LDA>2).

Supplementary Tables

Table S1 Demographic information for four bear species in Beijing zoo

Species	Individuals	Age	Gender
Giant Panda (*Ailuropoda melanoleuca*)	10	Juvenile:4	Male:5
		Adult: 6	Female:5
Brown Bear (*Ursus arctos*)	4	Juvenile:3	Male:3
		Adult: 1	Female:1
Polar Bear (*Ursus maritimus*)	4	Adult: 4	Male:2
			Female:2

Asian Black Bear (*Ursus thibetanus*)	3	Juvenile:1<br>Adult: 2	Male:1<br>Female:2

Table S2 The living schedule and diet of four types of bears.

Polar bear				Black bear and Brown bear				Panda			
**Feed Name**	**Weight (kg)**	**Main Nutritional Components**	**Quantity**	**Feed Name**	**Weight (kg)**	**Main Nutritional Components**	**Quantity**	**Feed Name**	**Weight (kg)**	**Main Nutritional Components**	**Quantity**
Beef	3.75	Protein	62.09%	Cornbread	2.5	Protein	14.33%	Cornbread	1.15	Protein	10.47%
Ox Heart	3	Fat	10.39%	Apple	2.15	Fat	3.35%	Apple	0.45	Fat	2.67%
Cornbread	1.25	Crude Fiber	0.94%	Carrot	7.15	Crude Fiber	6.29%	Carrot	0.5	Crude Fiber	35.44%
Apple	1.75	Carbohydrates	23.29%	Cucumber	1.45	Carbohydrates	77.60%	Bamboo Shoot	6	Carbohydrates	7.48%
Cucumber	1.5	Ash Content	4.23%	Pear	2.85	Ash Content	5.15%	Bamboo	15	Ash Content	6.16%
Tomato	0.55	Energy	131834.50KJ	Tomato	0.35	Energy	49992.41 KJ	Egg	0.2	Energy	153366 KJ
Rapeseed	1			Peach	1.45						
Carrot	0.6			Watermelon	3.2						
Watermelon	7.5			Beef	0.3						
Mackerel	15			Chicken Frame	0.3						

Table S3 The gene-specific primer sequences used for Real-time -qPCR.

**Primers**	**Oligonuncleotide sequence (5'-3')**	**Product size (bp)**
NPY (forward)	TCG CTC TGT CCC TGC TCG TGT G	116
NPY (reverse)	TCT CTT GCC GTA TCT CTG CCT GGT G	
AgRP (forward)	GCC CTG TTC CCA GAG TTC CC	114
AgRP (reverse)	ATC TAG GAC CTC CGC CAA AGG	
POMC (forward)	AAG ATG GGC TCT ACG GGA TG	134
POMC (reverse)	GTT CTT GAC GAT GGC GGT CT	
CART (forward)	TGG AAC CTG GCT TTA GCA AC	145
CART (reverse)	TAC TCT GCA CAT GCC GAC AC	
β-actin (forward)	TTG TGC GTG ACA TCA AAG AG	200
β-actin (reverse)	ATG CCA GAA GAT TCC ATA CC	

Table S4 The clean tags for each sample.

Sample_ID	clean_tags	valid_tags	valid_percent	valid minLength	valid meanLength	valid maxLength	OTU_counts	Total_OTUs
CB1	75883	64077	84.44%	258	413.53	442	999	26164
CB2	76438	63799	83.47%	259	414.21	442	1386	26164
CB3	77541	61218	78.95%	258	409.5	441	1239	26164
CB5	74760	61649	82.46%	258	407.69	441	1626	26164
CB6	74189	62758	84.59%	323	411.22	441	1578	26164
CH1	76557	67560	88.25%	258	418.9	442	1130	26164
CH2	76354	65104	85.27%	219	416.65	441	1336	26164
CH3	74906	68746	91.78%	258	408.29	441	1139	26164
CH4	78219	71006	90.78%	258	425.01	442	1000	26164
CP1	74983	68217	90.98%	248	424.49	442	757	26164
CP2	75940	72570	95.56%	219	425.44	442	773	26164
CP3	74293	71697	96.51%	258	425.55	442	752	26164
CP4	76313	71660	93.90%	258	425.19	442	786	26164
CP5	76065	68700	90.32%	258	424.77	442	808	26164
CP6	76950	68907	89.55%	258	417.21	441	749	26164
CP7	74707	66347	88.81%	258	424.94	442	951	26164
CP8	76327	73980	96.93%	258	425.53	444	735	26164
CP9	75878	69244	91.26%	248	425.44	442	853	26164
CZ1	75476	66170	87.67%	258	421.89	442	804	26164
CZ2	77930	67218	86.25%	258	421.73	441	786	26164
CZ3	75210	67551	89.82%	258	425.16	442	744	26164
CZ4	75016	69393	92.50%	398	423.17	442	1703	26164
CZ5	75208	67783	90.13%	219	422.74	442	1255	26164
CZ6	74581	64406	86.36%	258	425.01	441	1401	26164
DB1	75561	62997	83.37%	248	424.48	442	750	26164
DB2	76020	63532	83.57%	258	423.03	442	912	26164
DH1	77129	65889	85.43%	258	414.7	441	1073	26164
DH2	77680	65658	84.52%	258	417.27	442	1083	26164
DP1	76559	71048	92.80%	255	425.25	442	785	26164
DP2	77378	71509	92.42%	258	422.75	442	800	26164
DP3	77585	68355	88.10%	229	416.96	442	648	26164
DP4	76094	70748	92.97%	248	425.53	442	715	26164
DP5	76743	70290	91.59%	258	423.15	442	929	26164
DP6	74881	67481	90.12%	258	421.92	442	708	26164
DP7	75449	63626	84.33%	259	412.63	442	768	26164
DP8	76580	59504	77.70%	259	423.96	441	840	26164
DP9	75496	64976	86.07%	258	415.14	442	846	26164
DZ1	77318	72016	93.14%	248	416.48	442	1323	26164

DZ2	77268	69228	89.59%	258	417.04	442	824	26164
DZ3	75630	66328	87.70%	258	424.51	442	879	26164
DZ4	75053	63283	84.32%	397	423.55	442	1059	26164
DZ5	74506	63325	84.99%	258	415.73	441	962	26164
QB3	76162	65809	86.41%	258	422.42	441	791	26164
QB1	74698	59635	79.83%	258	417.82	442	1165	26164
QB2	77615	75113	96.78%	259	425.66	442	990	26164
QH1	74761	60682	81.17%	259	413.68	442	1480	26164
QH2	74873	70830	94.60%	398	424.06	441	1387	26164
QH3	77159	64097	83.07%	258	419.12	451	1421	26164
QP1	77150	61911	80.25%	258	423.14	441	997	26164
QP2	74833	68704	91.81%	248	424.72	442	802	26164
QP3	77033	69252	89.90%	258	424.11	442	796	26164
QP4	75787	64631	85.28%	258	415.91	442	820	26164
QP5	74545	66073	88.64%	259	419.53	442	946	26164
QP6	75161	67863	90.29%	323	415.21	441	873	26164
QP7	75992	63748	83.89%	259	419.78	441	969	26164
QP8	74350	63391	85.26%	259	419.3	441	954	26164
QP9	77550	64605	83.31%	248	420.33	442	1092	26164
QZ1	77369	69967	90.43%	259	425.43	442	859	26164
QZ2	75636	68927	91.13%	259	425.51	441	849	26164
QZ3	75353	71998	95.55%	248	425.61	441	809	26164
QZ4	75289	70584	93.75%	259	425.71	442	643	26164
XB1	76818	68199	88.78%	258	424.22	451	1151	26164
XB2	77630	65813	84.78%	258	414.25	442	1237	26164
XB3	76841	63377	82.48%	258	407.18	441	1508	26164
XB4	76229	63031	82.69%	258	414.68	442	1455	26164
XH1	75352	67453	89.52%	258	423.5	442	1224	26164
XH2	74069	70921	95.75%	259	425.07	442	1070	26164
XH3	74702	70041	93.76%	259	420.94	441	1048	26164
XP1	74685	67228	90.02%	248	425.67	441	843	26164
XP2	77512	72777	93.89%	400	425.1	441	898	26164
XP3	77559	64315	82.92%	248	425.53	441	880	26164
XP4	76206	72716	95.42%	220	425.58	442	802	26164
XP5	76639	69438	90.60%	258	425.72	442	772	26164
XP6	77585	70872	91.35%	258	424.81	441	885	26164
XP7	76464	65564	85.74%	259	416.08	442	935	26164
XP8	75728	68995	91.11%	296	421.83	442	877	26164
XP9	75321	63402	84.18%	248	423.79	442	907	26164
XZ1	74608	71280	95.54%	259	425.52	442	729	26164
XZ2	77246	69069	89.41%	248	422.5	442	744	26164

XZ3	77441	69612	89.89%	259	420.78	442	814	26164
XZ4	75577	70453	93.22%	400	425.04	442	818	26164

Table S5 The ANOSIM results of four bear species.

Group	distance	R	P_value	P_adj_BH
Polar/Black	Bray-Curtis	0.364056996	0.001	0.002
Polar/Panda	Bray-Curtis	0.5420409	0.001	0.002
Polar/Brown	Bray-Curtis	0.50903208	0.001	0.002
Black/Panda	Bray-Curtis	0.085921256	0.118	0.118
Black/Brown	Bray-Curtis	0.20294703	0.013	0.0156
Panda/Brown	Bray-Curtis	0.155335801	0.005	0.0075

Table S6 Comparison results of phyla pairwise of 4 bears in 4 seasons (Mann-Whitney *U* test, fdr_P< 0.05).

Microbe	Season	Bear1	Bear2	Statistic	p_value	fdr_corrected_p_value	power
Fusobacteriota	Spring	Panda	Polar	0	0.002797203	0.033566434	0.839085779
Fusobacteriota	Summer	Panda	Polar	0	0.002797203	0.033566434	0.823660795
Gemmatimonadota	Summer	Panda	Polar	0	0.002797203	0.038590448	0.81281937
Gemmatimonadota	Winter	Brown	Panda	45	0.003215871	0.038590448	0.839690595
Gemmatimonadota	Spring	Black	Panda	27	0.009090909	0.043636364	0.830966166
Gemmatimonadota	Summer	Black	Panda	27	0.009090909	0.043636364	0.995188542
Gemmatimonadota	Autumn	Black	Panda	27	0.009090909	0.043636364	0.996146754
Fusobacteriota	Spring	Brown	Polar	0	0.00952381	0.045714286	0.837997888
Fusobacteriota	Summer	Black	Panda	27	0.009090909	0.045714286	0.846297817
Fusobacteriota	Winter	Panda	Polar	0	0.009090909	0.045714286	0.803886459

Table S7 Comparison results of genus pairwise of 4 bears in 4 seasons (Mann-Whitney *U* test, fdr_P< 0.05).

Genus	Season	Bear1	Bear2	Statistic	p_value	fdr_corrected_p	power
F082	Autumn	Black	Panda	27	0.0042345	0.02328975	0.87872124
Lachnospiraceae_AC2044_group	Autumn	Black	Panda	27	0.010293155	0.038599331	0.835319378
Plesiomonas	Autumn	Brown	Panda	36	0.006547011	0.041103019	0.856937375
Escherichia.Shigella	Spring	Brown	Panda	54	0.0003996	0.00959041	0.893132468

Alistipes	Spring	Brown	Panda	54	0.0003996	0.00959041	0.847271419
Treponema	Spring	Black	Panda	27	0.0042345	0.0155265	0.835492003
Sarcina	Spring	Brown	Panda	51	0.002797203	0.016783217	0.817287779
Sarcina	Spring	Panda	Polar	0	0.002797203	0.016783217	0.832475926
Bacteroides	Spring	Brown	Panda	53	0.000799201	0.019180819	0.831852527
Alistipes	Spring	Panda	Polar	0	0.002797203	0.022377622	0.848287366
F082	Spring	Black	Panda	27	0.0042345	0.02328975	0.997924409
Streptococcus	Spring	Brown	Panda	2	0.001598402	0.033566434	0.819636726
Streptococcus	Spring	Panda	Polar	36	0.002797203	0.033566434	0.953751685
Klebsiella	Spring	Panda	Polar	0	0.002797203	0.033566434	0.823293088
Romboutsia	Spring	Brown	Panda	52	0.001598402	0.033566434	0.821504755
Romboutsia	Spring	Panda	Polar	0	0.002797203	0.033566434	0.801252133
Alistipes	Spring	Black	Panda	27	0.009090909	0.036363636	0.841809496
Lactobacillus	Spring	Brown	Panda	52	0.001598402	0.038361638	0.831695464
Plesiomonas	Spring	Brown	Panda	52	0.003278564	0.041103019	0.829551501
Plesiomonas	Spring	Panda	Polar	0	0.005474564	0.041103019	0.807123533
Romboutsia	Spring	Black	Panda	27	0.009090909	0.043636364	0.843715974
Klebsiella	Spring	Brown	Polar	0	0.00952381	0.045714286	0.812068746
Plesiomonas	Spring	Brown	Polar	0	0.00952381	0.045714286	0.802227419
Streptococcus	Spring	Black	Panda	0	0.009090909	0.047952048	0.89868795
Streptococcus	Spring	Brown	Polar	24	0.00952381	0.047952048	0.892477531
Treponema	Summer	Black	Panda	27	0.0042345	0.0155265	0.926194528
Treponema	Summer	Panda	Polar	0	0.002078183	0.0155265	0.847292142
Sarcina	Summer	Brown	Panda	36	0.002797203	0.016783217	0.834153915
Lachnospiraceae_AC2044_group	Summer	Black	Panda	27	0.0042345	0.0211725	0.935206536
Lachnospiraceae_AC2044_group	Summer	Panda	Polar	0	0.002078183	0.0211725	0.856922846
Alistipes	Summer	Panda	Polar	0	0.002797203	0.022377622	0.905292049
Sarcina	Summer	Panda	Polar	1	0.005594406	0.026853147	0.828816699
Sarcina	Summer	Black	Panda	27	0.009090909	0.027272727	0.804021346
Muribaculaceae	Summer	Brown	Panda	0	0.002797203	0.033566434	0.83156842
Muribaculaceae	Summer	Panda	Polar	0	0.002797203	0.033566434	0.862746137
Plesiomonas	Summer	Panda	Polar	0	0.006850503	0.041103019	0.828646434
Streptococcus	Summer	Panda	Polar	34	0.011188811	0.047952048	0.815223507
Sarcina	Winter	Brown	Panda	45	0.000999001	0.016783217	0.810624327
Lachnospiraceae_AC2044_group	Winter	Black	Panda	27	0.0042345	0.0211725	0.801294436
Klebsiella	Winter	Brown	Panda	45	0.000999001	0.023976024	0.830394263
Alistipes	Winter	Brown	Panda	43	0.003996004	0.023976024	0.807534178
Sarcina	Winter	Black	Panda	27	0.009090909	0.027272727	0.806158746
Sarcina	Winter	Panda	Polar	0	0.009090909	0.027272727	0.805750394
Alistipes	Winter	Black	Panda	27	0.009090909	0.036363636	0.880174621
Romboutsia	Winter	Black	Panda	27	0.009090909	0.043636364	0.807665331

Romboutsia	Winter	Panda	Polar	0	0.009090909	0.043636364	0.807873846
Klebsiella	Winter	Black	Panda	27	0.009090909	0.045714286	0.909439074
Klebsiella	Winter	Panda	Polar	0	0.009090909	0.045714286	0.817398613
Streptococcus	Winter	Brown	Panda	4	0.011988012	0.047952048	0.854111208

Table S8 The ANOSIM results of winter FMT groups.

group	distance	R	P_value	P_adj_BH
Polar-FMTw/Control	Bray-Curtis	0.226666667	0.087	0.096666667
Polar-FMTw/Black-FMTw	Bray-Curtis	0.265873016	0.076	0.096666667
Polar-FMTw/Panda-FMTw	Bray-Curtis	0.266666667	0.028	0.093333333
Polar-FMTw/Brown-FMTw	Bray-Curtis	0.189333333	0.065	0.096666667
Control/Black-FMTw	Bray-Curtis	0.325	0.087	0.096666667
Control/Panda-FMTw	Bray-Curtis	0.173333333	0.079	0.096666667
Control/Brown-FMTw	Bray-Curtis	0.396	0.004	0.04
Black-FMTw/Panda-FMTw	Bray-Curtis	0.261904762	0.05	0.096666667
Black-FMTw/Brown-FMTw	Bray-Curtis	0.0375	0.393	0.393
Panda-FMTw/Brown-FMTw	Bray-Curtis	0.434666667	0.019	0.093333333

Table S9 The ANOSIM results of summer FMT groups.

group	distance	R	P_value	P_adj_BH
Polar-FMTS/Control	Bray-Curtis	0.637512148	0.002	0.01
Polar-FMTS/Black-FMTS	Bray-Curtis	0.361516035	0.011	0.021428571
Polar-FMTS/Panda-FMTS	Bray-Curtis	0.223517979	0.02	0.025
Polar-FMTS/Brown-FMTS	Bray-Curtis	0.407191448	0.004	0.013333333
Control/Black-FMTS	Bray-Curtis	0.271137026	0.015	0.021428571
Control/Panda-FMTS	Bray-Curtis	0.350826045	0.014	0.021428571
Control/Brown-FMTS	Bray-Curtis	0.491739553	0.002	0.01
Black-FMTS/Panda-FMTS	Bray-Curtis	0.095238095	0.11	0.11
Black-FMTS/Brown-FMTS	Bray-Curtis	0.247813411	0.013	0.021428571
Panda-FMTS/Brown-FMTS	Bray-Curtis	0.137026239	0.076	0.084444444
